# Policy, Theory, and Evaluation: Stop Mixing the Fruit Salad: Comment on "Developing a Framework for a Program Theory-Based Approach to Evaluating Policy Processes and Outcomes: Health in All Policies in South Australia"

**DOI:** 10.15171/ijhpm.2018.35

**Published:** 2018-04-17

**Authors:** Evelyne de Leeuw

**Affiliations:** ^1^Centre for Health Equity Training Research and Evaluation (CHETRE), University of New South Wales, Sydney, NSW, Australia.; ^2^South Western Sydney Local Health District, Liverpool, NSW, Australia.; ^3^Ingham Institute, Liverpool, NSW, Australia.

**Keywords:** Policy, Power, Theory, Evaluation, Joined-Up Government

## Abstract

The study of Health in All Policies (HiAP) is gaining momentum. Authors are increasingly turning to wide swathes of political and social theory to frame (Program) Theory Based (or Informed) Evaluation (TBE) approaches. TBE for HiAP is not only prudent, it adds a level of elegance and insight to the research toolbox. However, it is still necessary to organize theoretical thinking appropriately. A commentary on a recent *Int J Health Policy Manag* paper argued that the framing of context and causality were hard to establish. This paper argues that this is not the most pressing issue. Rather, it claims we need to go back to basics to establish an appropriate HiAP evaluation paradigm. Such a basic paradigm would hinge on an understanding of power.

A good theory is as practical a tool any evaluator could wish for. Theory is a functional but limited model of a phenomenon in the real world. It provides the researcher with a lens to make sense of otherwise complex and messy issues.

For instance, in classical mechanics Newton’s law of universal gravitation (the second law of motion: F = ma) states that a particle attracts every other particle in the universe with a force which is directly proportional to the product of their masses and inversely proportional to the square of the distance between their centers. The theory allows us to predict how quickly an apple will fall, or what the ballistic trajectory of a cannon ball (or suborbital rocket) would be.

However, *how* this gravitation exists was even beyond Sir Isaac at the time (read his third letter to Bishop Richard Bentley^1^) – this became the territory of modern quantum physics.

Theories are useful to a particular purpose. Newton’s law allowed for the calculation of motion, not for understanding the distortion of spacetime. Similarly we, in the 21st century, continue to be hygienic about our choice of explanatory model for the phenomena we are investigating. Theories from the psychology domain about behavior change may not necessarily provide good insight into, for instance, the politics of morality. With some mental force and creativity such – almost neighboring – theoretical domains can be made to fit the issue under study (either behavior, or morality), but one would be better off having a clear conceptual match. A good abstraction of reality provides readily discoverable research parameters (F, m, and a, in the case of Newton, for Force, mass, and acceleration) and the ways to identify and measure them. The measurement and prediction of objects falling or ballistic paths does not require a classification of color, attitude, or altruism. It simply needs three measures for F, m and a.

In an age of post-modern social constructivism such an assertion more often than not creates cognitive pain and a flight forward into eclecticism, rather than a search for theories that might actually work for you. A theoretical nihilism is spreading like the proverbial miasma and colleagues around the world proudly avow that ‘anything goes’ or that ‘I don’t need theory!’ For instance, in the field of the health sciences we have found that most policy research is devoid of theory^2^ and the first bits of strong theory have yet to be applied to an issue that is deemed critical to population health these days, health literacy.

One must, however, also acknowledge the enormity of the task to identify the issue one is researching, and link that to a perfectly matching theory that would drive the research process. All too often I have met with budding researchers who wished to evaluate a particular intervention in a particular neighborhood and who then enthusiastically claimed ‘*Well, no-one has ever published a theory about local playgrounds in the Wittevrouwenveld neighborhood! Now I will have to do qualitative research, perhaps a focus group or two, to gauge what people think about playgrounds there*.’ It is up to the somewhat more mature (one would hope), possibly wiser and certainly sadder professor to explain that appropriate theories might well be available but that the issue needs to be morphed and reframed into one that matters. In this example one might wonder whether the presence of activity hardware necessarily leads to increased usage of the material, and if so (or if not) whether this might be different for different kinds of kids.^3^

So – theory is a practical tool. Not every issue needs to be explored with Grounded Theory (an approach to framing emergent insights for research purposes^4^). In many cases a fairly straightforward theory is already available and can be applied. This was the premise for Birckmayer and Weiss’ assertion that the best evaluations are theory-based,^5^ so nicely reframed for the health promotion field by Van den Broucke.^6^ Theory-based evaluation (TBE) yields superior insights, not just for research, but for intervention development, too.^7^

It is therefore absolutely pleasing to see the paper by Lawless et al^8^ adopting this approach. They describe the process they followed for establishing TBE parameters looking at the Health in All Policies (HiAP) programme of work that was so successfully started in the state of South Australia. In the evolution of this process they iteratively engaged with the policy process as they witnessed it first-hand, as well as their own team of researchers-practitioners who benefitted from a longer term progression of insight. I am reminded of recent work by policy science-guru Michael Howlett and his colleagues McConnell and Perl^9^ in which they describe the policy process as weaving a fabric – and in the case of the South Australians with a pattern of evaluation woven into the cloth. At least this is a better metaphor than the juggling one we proposed earlier^10^: policy, for Howlett and colleagues and Lawless and their partners, is tangible output rather than a nice nimble-fingered circus act….

But in Peña’s commentary^11^ to the Lawless et al paper the eclectic and multi-theory informed approach is criticized for being too complex, and for not establishing a clear causal logic. One would be tempted to regard this critique as cheap rhetoric. HiAP by necessity are complex and require complex explanatory heuristics, and as we have asserted elsewhere^12^ policy-making is a messy affair with constantly changing causalities and simultaneous iterations of phases that in a more traditional ‘stages heuristic’ were considered sequential to each other. For instance, the realities of politics dictate a preference for certain *types* of implementation paths even before a policy process logic would neatly dictate what would be the academically ‘best’ implementation approach. Lawless et al do not stand alone; a similar piece of work was published earlier in the *Annual Reviews.*^13^

But then again – is it perhaps too cowardly a way out to claim that HiAP is a mess (or rather, something that requires ‘complexity thinking’) and that we require an overturned fruit basket of theories to make sense of it…? Is there no other way that allows us to make sense of all this? Couldn’t we find a few common parameters that are the same for any HiAP-type approach that could – or should – be exposed to an existing theoretical heuristic?

Reflecting on this, my thinking boils down to two key premises. The first is what Peters has called the Holy Grail of (the science of) public administration^14^ – coherence and integration of public policy and the organizational parameters to achieve it. The second hinges on the belief that policies, (sociological and hardware) institutions, and aspirations are all ultimately created and sustained by (groups of) people – be they communities, interest groups or politicians and bureaucrats. *Any* research into HiAPs should start with the adoption of an internally consistent theoretical proposition that those groups of people have their reasons to either clarify clear paths to resolving complex issues, or to continue to obfuscate and frustrate those matters. At its most simple: this is a matter of power.

One would be tempted to step away from the eclectic postmodern social constructivist approach to understanding complex social and policy processes, and simply go for Robert Dahl’s pluralist theory of democracy^15^ – hinging on a deep and concrete understanding of power, its sources and its consequences. Dahl prompts us to think about this by describing the sources of power as:

“*An individual’s own time; access to money, credit and wealth; control over information; esteem or social standing; the possession of charisma, popularity, legitimacy, legality; the rights pertaining to public office; solidarity: the capacity of a member of one segment of society to evoke support from others who identify him (her) as like themselves because of similarities in occupation, social standing, religion, ethnic origin, or racial stock; the right to vote; intelligence; education; and perhaps even one’s energy level.*”

To the budding health policy researcher such a perspective on the power question might be a bit overwhelming. And of course it is. The question of power may be at the very heart of the human spirit and at the continuing quest of many of my colleagues (and myself) for health equity. Indeed, many a philosopher has dwelt on the issue. The famous contemporary philosopher Beyoncé Knowles^16^ has stated that “*Power’s not given to you. You have to take it*” echoing a sentiment by revolutionary Che Guevara:

*“Power is the sine qua non strategic objective of the revolutionary forces, and everything must be subordinated to this basic endeavor. But the taking of power, in this world polarized by two forces of extreme disparity and absolutely incompatible in interests, cannot be limited to the boundaries of a single geographic or social unit. The seizure of power is a worldwide objective of the revolutionary forces. To conquer the future is the strategic element of revolution; freezing the present is the counterstrategy motivating the forces of world reaction today, for they are on the defensive.”* ^17^

The political scientist Dahl and others like Charles Lindblom and Dennis Wrong^18^ present an understandably more sobering view than the glorious empowerment assertions for a world of haves and have-nots, be they women (Knowles) or the proletariat (Guevara). I am not intending to provide a primer in power studies and concepts; the idea of power can be as simple as Wrong’s definition *‘…the capacity of some persons to produce intended and foreseen effects on others*’ and as complex as a systemically imbued version of Dahl’s perspective above: not individuals, but often abstract systems and beliefs (‘institutions’ as per Hannah Arendt’s views on violence – and mediocrity^19^) create and sustain the patterns and mechanisms of power – and its consequences. But to claim that the underling must *take* power is not my understanding how things work. Mostly, we underlings simply are not exploiting the sources of power we still have – often by default. First of all, power is given by the subordinate person or system to the superior (eg, if you *believe* or accept that someone has power over you it has already become a given…). Often this happens because the subordinate does not recognize that he, she, or it, may have yielded the sources of power unwittingly (eg, through forfeiting the right to vote in a referendum, say, as a young Brit to stay in, or leave, the European Union). It may also happen that groups in society, through often rather perverse mechanisms such as racism or heteronormativity, self-impose limitations to their degrees of freedom and are unable to seize their own sources of power. These sources could well be sheer numbers and the capacity to speak, or stop (in the middle of a road, work, listening, or complying with social or occupational standards – think of Gandhi’s nonviolent resistance^20^).

The giving or taking of power, and understanding individual and systems options in doing so, is critical to our understanding of the conditions under which HiAP may be shaped, implemented, and impact on health and health equity. For instance, in thinking about some of the foundational considerations for HiAP (and its predecessor, Healthy Public Policy) it only dawned on me quite recently^13^ (and after a 30-year career pondering these questions) that HiAP researchers, lobbyists and practitioners may be doing themselves the greatest disservice of all placing themselves in the same part of the playing field as the public (sector) health bureaucracy. Of course, most of these HiAP persons will have a background in a health discipline. Surely, they believe that health is of pre-eminent importance to all of humanity. But to then assume that such lofty ideals will be shared without any cynical hesitation by the health care system and its bureaucracy is dangerously self-delusional. The health care system, driven by medical-industrial interests, has very little to *win* (now think about the sources and concepts of power outlined above) embracing complexity and joined-up government. In fact – and I see this every day in Australia – it has everything to win by maintaining fragmentation (between levels of government, for instance) and sustaining incoherent control (say, of financial management of the health care purse).

So let’s take the healthcare system and its bureaucracies out of the theoretical equation. Would we not be able to explain why and how complex health issues are framed in joined-up HiAP solutions by starting to take a look at the sources of power and their distribution among the particular configurations of stakeholders around the issues that are deemed ‘amenable’ to HiAP-like solutions…? Once we have described that baseline landscape I bet we would more easily identify, and better suit, sets of theories like those used by Lawless and colleagues and start explaining and predicting more optimal ways forward. Yes – there is context, and yes, there is messy causality. But we are challenged to make sense of context and causality before we start compiling a multidimensional theoretical hybrid.

In sum: we need TBE to make sense of HiAP. A multitude of theoretical gazes and conceptual heuristics is possible. But I would argue we start with the basics before we mix all of them into a fruit salad.

## Ethical issues

Not applicable.

## Competing interests

Author declares that she has no competing interests.

## Author’s contribution

EdL is the single author of the paper.
